# Salivary duct carcinoma presenting with unilateral multiple cranial nerve lesions and concurrent intracranial metastasis: A case report

**DOI:** 10.1097/MD.0000000000041293

**Published:** 2025-02-07

**Authors:** Bo Zhu, Qing-Zi Zhang, Kun Guo, Nai-Bing Gu, Zheng-Li Di, Zhi-Qin Liu, Xiang-Chun Yang, Li-Li Chai, Yan-Ping Yang, Xiao-Tao Jia

**Affiliations:** a Department of Neurology, The Affiliated Xi’an Central Hospital of Xi’an University College of Medicine, Xi’an, Shaanxi, People’s Republic of China; b Yan’an University School of Medicine, Yan’an, Shaanxi, People’s Republic of China; c Department of Imaging, The Affiliated Xi’an Central Hospital of Xi’an University College of Medicine, Xi’an, Shaanxi, People’s Republic of China; d Department of Patnology, The Affiliated Xi’an Central Hospital of Xi’an University College of Medicine, Xi’an, Shaanxi, People’s Republic of China; e Department of Neurosurgery, The Affiliated Xi’an Central Hospital of Xi’an University College of Medicine, Xi’an, Shaanxi, People’s Republic of China.

**Keywords:** intracranial metastatic tumors, multiple cranial nerve lesions, salivary duct carcinoma

## Abstract

**Rationale:**

Salivary gland ductal carcinoma (SDC) is a rare malignant tumor of the head and neck, accounting for 1% to 3% of salivary gland malignancy. SDC usually occurs in parotid gland, has a high malignancy degree, poor prognosis, and is prone to distant metastasis. In particular, intracranial metastasis accompanied by multiple cranial nerve lesions is very rare, and early diagnosis is difficult, which easily leads to missed diagnosis and misdiagnosis. Therefore, the diagnosis and treatment of SDC face major challenges. We report a unique case of SDC, which initially presented with progressive facial paralysis followed by progressive intracranial metastases and multiple cranial nerve lesions. After surgery and pathological examination, SDC was finally diagnosed.

**Patient concerns:**

A previously healthy 53-year-old male with persistent facial paralysis gradually developed symptoms of ipsilateral multiple cranial nerve lesions, and was hospitalized 8 months after onset.

**Diagnoses:**

Brain magnetic resonance imaging enhancement showed multiple irregular intracranial T1 and T2 with uneven signal intensity. Brain computed tomography showed intracranial space-occupying lesions, the largest of which was about 55 × 42 × 38 mm, with extensive bone destruction. Pathological findings showed malignant cell mass in the left temporal lobe tumor. Immunohistochemical: (++), CK7(+++), AR(++), Brg-1(+), Her-2(2+), Ki67(+, approximately 80%), consistent with high-grade ductal carcinoma, considering salivary gland origin. The final diagnosis was SDC.

**Interventions:**

The space-occupying lesions in the left cavernous sinus region and temporal lobe were partially resected. Local radiotherapy was recommended, but the patient refused.

**Outcomes:**

Postoperative symptoms did not improve significantly. Follow-up after 4 months showed no improvement in the extensive cranial nerve lesions on the left side.

**Lessons:**

In clinical practice, in the face of persistent facial palsy symptoms that gradually spread to other cranial nerves, the possibility of secondary facial nerve injury and malignant salivary gland tumors, especially SDC, cannot be easily ruled out even if imaging studies do not reveal any space-occupying intracranial lesions. Through the comprehensive use of imaging, pathology and immunohistochemistry and other diagnostic methods, the early detection and accurate diagnosis of SDC can be achieved, so as to provide the best treatment strategy for patients and improve the prognosis.

## 1. Introduction

Diffuse multiple cranial nerve damage, especially when associated with intracranial space-occupying lesions, is extremely rare. It suggests the possibility of potential head and neck tumor, which can be easily misdiagnosed in the early stages. SDC is a rare malignant tumor of the head and neck. It typically affects only single cranial nerve, with diffuse involvement being even rare. This article reports a case of progressive facial nerve palsy, which was followed by the gradual onset of multiple cranial nerve lesions and destruction of the skull base, confirmed postoperatively as SDC.

## 2. Case report

A 53-year-old man with no significant past medical history initially presented with persistent unilateral facial paralysis and gradually developed symptoms of multiple cranial nerve lesions, and was admitted to the hospital in January 2024. Eight months ago, the patient begin presented with incomplete closure of the left eye fissure and rightward deviation of the corner of the mouth. The Brain magnetic resonance imaging (MRI) showed no significant abnormalities, so this condition was considered “idiopathic facial nerve palsy,” but neurotrophic therapy was ineffective and the condition progressively worsened. During the next 6 months, the patient developed ptosis of the left eyelid, inability to frown on the left, left corner of the mouth drooping, tongue deviation to the right, and difficulty moving to the right. Subsequently, hoarseness, dysphagia, blurred vision, decreased hearing on the left, pronounced dizziness, and recurrent hiccups developed. Electronic laryngoscope examination showed the left vocal cord is immobilized. A 3-dimensional computed tomography (CT) imaging of the facial nerve showed enlargement of labyrinthine and tympanic segments on the left, with thinning of the adjacent tympanic bone, and the symptomatic treatment was poor, leading the patient to seek diagnosis and treatment at our hospital. Occasional pain on the left facial and cranial regions in the past year. Sometimes touched the left mandibular “peanut” large lump. Weight loss of about 10 kg.

**Figure 1.**
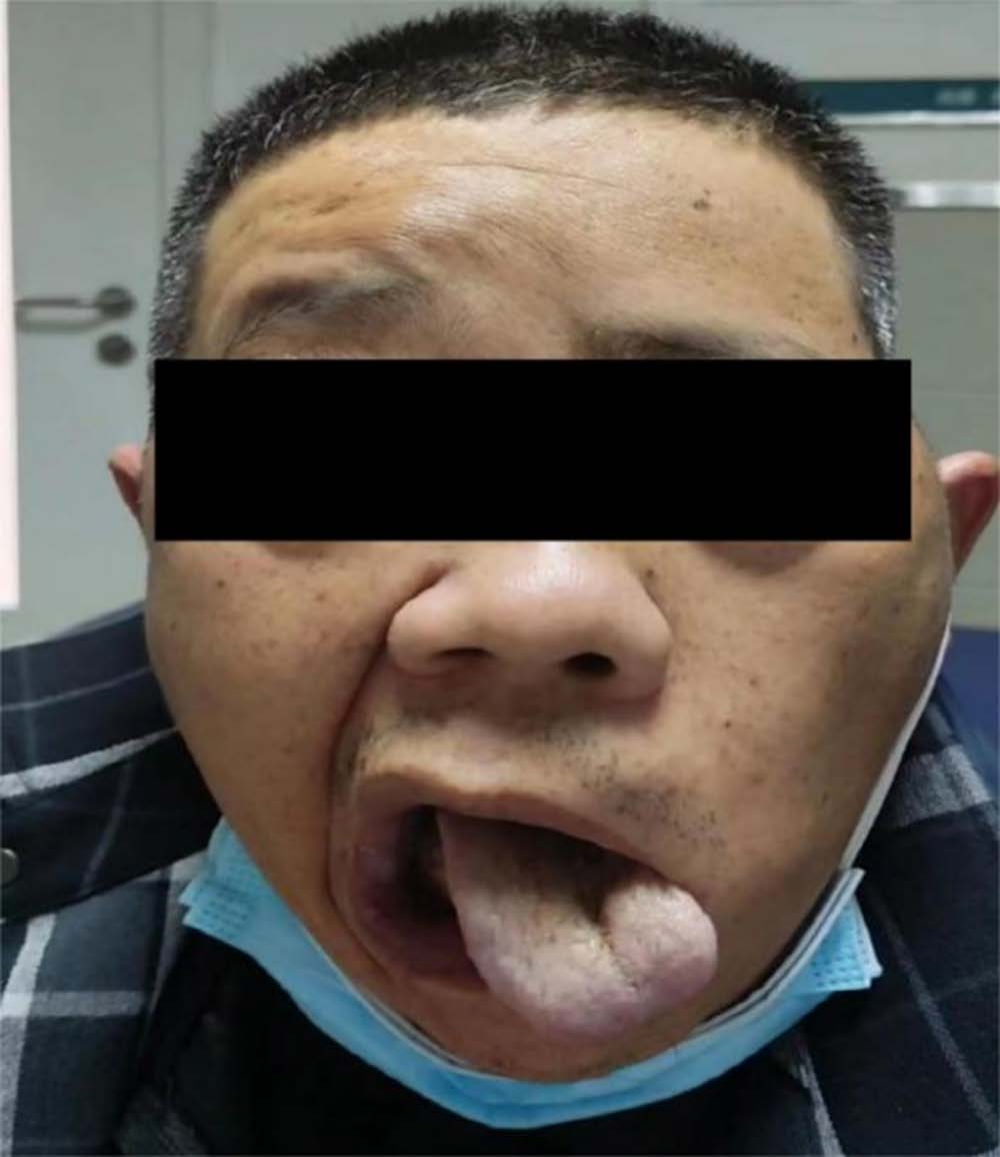
The left face of patient reduced frontalis wrinkling, shallower nasolabial fold, corner of the mouth deviation to right, tongue deviation to the right and tongue muscles marked atrophy.

Physical examination revealed a clear mind, dysarthria; a fixed, hard, enlarged lymph node measuring 3 cm × 2 cm in the left submandibular area; decreased vision on the left; ptosis of the left eyelid, slight proptosis of the left eyeball, adduction state, dilated pupil, irregular, with a diameter of 5mm, with sluggish light reflex; drooply sensation in the left ophthalmic and maxillary branches, reduced frontalis wrinkling on the left, shallower nasolabial fold on the left, corner of the mouth deviation to right, left hearing loss, left tongue muscles marked atrophy, tongue deviation to the right, shoulder hunching and neck rotation left weak (Fig. 1).

**Figure 2.**
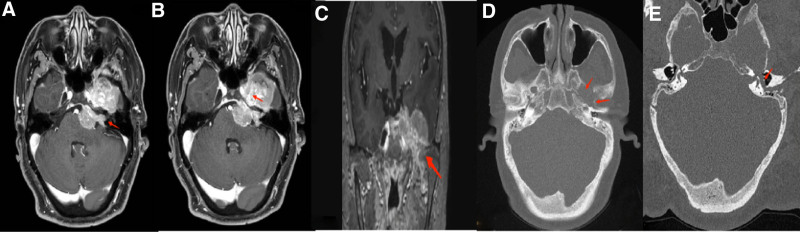
(A) Brain MRI (enhanced) showed involvement of the left internal auditory canal. (B) Brain MRI (enhanced) showed involvement of cavernous sinus. (C) Brain MRI (enhanced) showed enlargement of Left foramen ovale and involvement of mandibular nerve. (D) Brain CT showed destruction and enlargement of left foramen ovale and spinous. (E) Brain CT showed destruction and enlargement of left facial canal. CT = computer tomography, MRI = magnetic resonance imaging.

Enhanced brain MRI revealed multiple irregular longer T1 and equal/longer T2 in the left internal auditory canal, cerebellopontine angle, cavernous sinus, temporal lobe adjacent to the tentorium, infratemporal fossa, oculomotor, trigeminal and facial nerves at the fundus of the internal auditory canal, geniculate ganglion, labyrinthine, tympanic and mastoid segments, with uneven signal intensity (Fig. 2A–C). Brain CT showed multiple irregular, slightly hyperdense masses in the left internal auditory canal, cerebellopontine angle, parasellar cavernous sinus, temporal lobe adjacent to the tentorium, infratemporal fossa and orbital apex, with the largest lesion measuring approximately 55 × 42 × 38 mm, and extensive bone destruction (Fig. 2D and E). Neck ultrasound revealed a hypoechoic mass within the left submandibular gland. Chest, abdominal, and pelvic CT showed no significant abnormalities. Pre-transfusion tests and tumor markers were within normal limits. Given the high likelihood of a malignant tumor, the large size of the lesion, and significant compression of the brainstem, the patient was taken to surgery for a partial resection, and the specimen was sent for examination.

**Figure 3.**
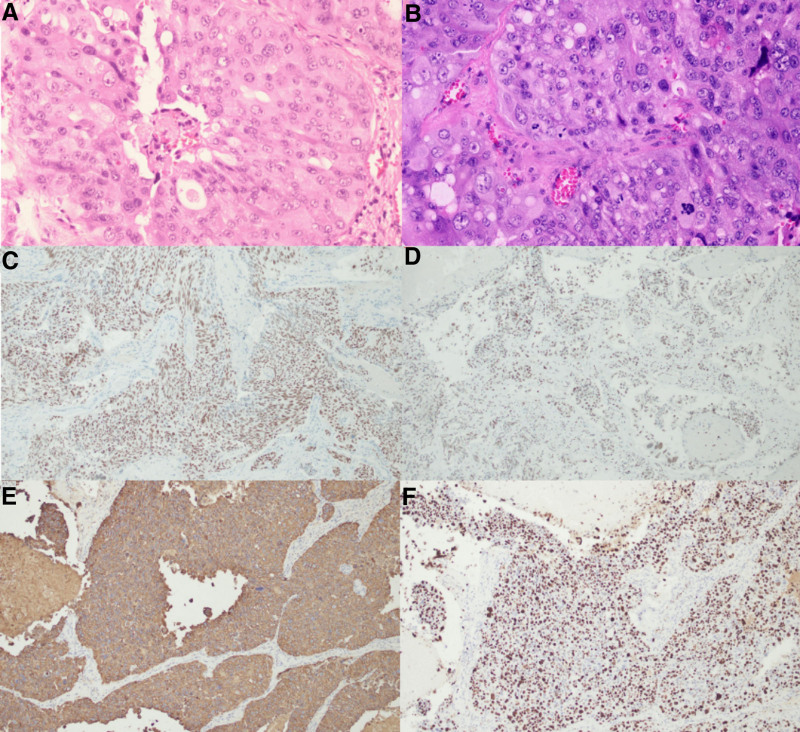
(A and B) Histopathological findings showing the polygonal cells with elliptical nuclei, prominent nucleoli, and eosinophilic cytoplasm. (C) Immunohistochemistry of tumor cells showing positive staining for AR. (D) Immunohistochemical results showing positivity in tumor cells for Brg-1. (E) Immunohistochemical results showing positivity in tumor cells for CK7. (F) Immunohistochemical results showing positivity in tumor cells for Ki67. (A and B) hematoxylin–eosin stain × 100, (C–F) immunohistochemistry × 100.

Pathological findings showed malignant cell mass in the left temporal lobe tumor. Immunohistochemical: EMA(++), CK7(+++), AR(++), Brg-1(+), Her-2(2+), Ki67(+, approximately 80%), consistent with high-grade ductal carcinoma, considering salivary gland origin (Fig. 3A–F). Local radiation therapy was recommended, but the patient refused. Follow-up after 4 months showed no improvement in the extensive cranial nerve lesions on the left side.

## 3. Discussion

SDC is a rare, highly malignant neoplasm of the head and neck with a poor prognosis, over 20 histological subtypes, accounting for approximately 1% to 3% of malignant salivary gland tumors, most common in the parotid gland, followed by the submandibular gland, with fewer occurrences in minor salivary glands.^[1]^ Facial paralysis is often the initial symptom of SDC. The most common cause of facial paralysis is idiopathic Bell palsy, however, malignant tumors account for about 5%, tumor invasion or compression of the facial nerve is often confused with Bell palsy.^[2]^ Viral-induced Bell palsy is characterized on MRI by multi-segmental, continuous linear enhancement of the facial nerve. The diagnosis requires not only typical clinical presentation but also the exclusion of other causes. In contrast, malignant tumors often present with focal enhancement. The patient presented with facial paralysis as the initial symptom, which was initially misdiagnosed as Bell palsy, condition persisted without resolution and gradually involved the left cranial nerves II–XII. The possibility of a malignant tumor was still overlooked during the medical consultation process, leading to rapid progression of the disease.

With the development of imaging, diseases causing multiple cranial nerve lesions are more and more easily detected. However, SDC initially only involves the facial nerve, and involvement of unilateral multiple cranial nerve is very rare, which was difficult to detect in early imaging.^[3]^ This is due to the fact that perineural invasion takes time to become apparent on imaging studies, also 1 of the reasons for missed diagnoses and delayed treatment.^[4]^ Garcin–Guillain syndrome is a common syndrome of unilateral multiple cranial nerve lesions, usually involving more than 7 pairs of cranial nerves. There are generally no symptoms of brain parenchymal damage, but may be extensive bone destruction of the skull base.^[5]^ Current research on unilateral multiple cranial nerve lesions are mostly reported in case reports, and there are no large-scale studies as of yet. Tumors and intracranial infections are common causes of unilateral multiple cranial nerve lesions. Primary central nervous system lymphoma is typically confined to the brain parenchyma, rarely spreads outside the central nervous system, may be associated with polycranial neuropathy, and high protein levels in the cerebrospinal fluid.^[6]^ Ramsay Hunt syndrome, induced by herpes zoster, can manifest as painful ophthalmoplegia and multiple cranial nerve involvement. The characteristical symptoms are peripheral facial paralysis, ear pain and ear herpes.^[7]^ Based on clinical symptoms and signs, it is indicated that cranial nerves II through XII are involved. Combined with the imaging, the metastatic pathway is predominantly considered to be direct invasion of the facial nerve by a tumor of the left submandibular gland, with extension along the internal auditory canal to involve the auditory nerve, and simultaneous inward erosion of the temporal bone, leading to rapid growth within tissue planes, compression of the cavernous sinus causing cavernous sinus syndrome, compression of the optic, oculomotor, trochlear, trigeminal, and abducens nerves. It spreads down to the jugular foramen along the petro-occipital fissure and compresses the glossopharyngeal, accessory, and vagus nerve. At the same time, it invaded the hypoglossal canal, affected the hypoglossal nerve. It spreads upward along the pore fissure to the hypoglossal canal, forming intracranial space.^[8]^ Although unilateral multiple cranial nerve lesions caused by SDC is rare, it should not be overlooked.

Among salivary gland tumors, SDC has the highest rate of distant metastasis, with female patients exhibiting higher survival rates than males. The most common sites of metastasis are the lungs, liver, and bones, with cerebral metastasis occurring in only 7% of cases, which also have a poorer prognosis.^[9]^ Brain metastasis of malignant salivary gland tumors typically occurs later, often following metastasis to the lungs and bones, with direct invasion of the cranium being even rarer.^[10]^ Furthermore, there are few case reports on the metastasis of malignant salivary gland tumors to the posterior cranial fossa, which may present as unilateral or multiple cranial neuropathies. The radiological features are similar to those of meningiomas or schwannomas, often leading to misdiagnosis.^[11]^ In this case, imaging studies suggested a presentation akin to “multiple schwannomas,” whereas histopathological findings confirmed SDC. Chest, abdomen, and pelvis CT revealed no evidence of metastatic lesions, with metastasis confined to the cranium, which is indeed a rare occurrence. In this case, the patient exhibited a large intracranial metastatic lesion with significant cerebral edema and brainstem compression, and there was no notable improvement in symptoms post-surgery. Follow-up 3 months post-surgery revealed that the patient continued to experience blurred vision, incomplete closure of the left eyelid, dysphagia, hoarseness, and hearing loss, severely impacting the quality of life. Due to the scarcity of large-scale clinical studies on intracranial metastasis of malignant salivary gland tumors, and the lack of specific clinical and radiological manifestations, misdiagnosis and missed diagnosis are common. This case provides a significant addition to the understanding of intracranial metastasis in SDC.

The early atypical clinical manifestations and imaging features of SDC are often easy to be missed and misdiagnosed. Although this patient is a classic case of salivary gland ductal carcinoma with multiple cranial nerve lesions, he only showed facial palsy symptoms in the early stage, which was difficult to distinguish from idiopathic facial palsy, resulting in missed diagnosis and affecting the patient’s prognosis. The imaging characteristics of salivary gland tumors are similar to those of meningiomas and schwannomas, which is a major challenge for clinicians and radiologists. Given that the diagnosis, treatment, and follow-up of this condition involve multiple specialties, including neurology, dentistry, otorhinolaryngology, neurosurgery, and radiology, multidisciplinary collaboration is essential for early diagnosis and treatment of the patient.

## 4. Conclusion

In clinical practice, in the face of persistent facial palsy symptoms that gradually spread to other cranial nerves, the possibility of secondary facial nerve injury and malignant salivary gland tumors, especially SDC, cannot be easily ruled out even if imaging studies do not reveal any space-occupying intracranial lesions. Through the comprehensive use of imaging, pathology and immunohistochemistry and other diagnostic methods, the early detection and accurate diagnosis of SDC can be achieved, so as to provide the best treatment strategy for patients and improve the prognosis.

## Author contributions

**Conceptualization:** Bo Zhu, Xiao-Tao Jia.

**Investigation:** Qing-Zi Zhang, Kun Guo, Xiang-Chun Yang, Li-Li Chai, Yan-Ping Yang.

**Writing – original draft:** Bo Zhu.

**Writing – review & editing:** Nai-Bing Gu, Zheng-Li Di, Zhi-Qin Liu, Xiao-Tao Jia.
